# Complete genome sequences of biocontrol agents *Serratia rubidaea* strains SY163 and SY183 effective against tomato gray mold

**DOI:** 10.1128/mra.01362-24

**Published:** 2025-05-19

**Authors:** Ryo Morimoto, Tomoko Hira, Takuji Oka, Daisuke Hira

**Affiliations:** 1 Department of Biotechnology and Life Sciences, Faculty of Biotechnology and Life Sciences, Sojo University 67181, Kumamoto, Japan; 2 Hira Labo, Kumamoto, Japan; University of Strathclyde 3527, Glasgow, United Kingdom

**Keywords:** biocontrol, *Serratia rubidaea*, complete genome

## Abstract

We report the genome sequences of *Serratia rubidaea* strains SY163 and SY183, isolated from tomato leaves using methanol as the sole carbon source. These strains are known to inhibit the gray mold pathogen, *Botrytis cinerea*. PacBio Sequel IIe sequencing yielded circular chromosomes of 4,915,045 and 4,978,521 bp, both with 59.1% G + C content.

## ANNOUNCEMENT

Gray mold, caused by *Botrytis cinerea*, poses a significant threat to tomato production worldwide, with resistant strains diminishing the effectiveness of traditional chemical control methods (1). Plant-associated symbiotic bacteria residing on leaves show promise as biocontrol agents because of their ability to produce antifungal compounds (2). In our previous study, *Serratia rubidaea* strains SY163 and SY183, isolated from tomato leaves using methanol as the sole carbon source, demonstrated substantial antifungal activity against *B. cinerea* (3). Herein, we present the complete genome sequences of these strains to facilitate further research into their biocontrol mechanisms.

Microbial samples were collected from tomato leaves grown in plastic greenhouses (130°38′21.68″E, 32°43′42.599″N, Kumamoto, Japan) on 1 July 2020. Cells of strains SY163 and SY183, stored as glycerol stocks in the laboratory of Sojo University, were cultured overnight at 30°C in Luria-Bertani medium under aerobic conditions. Genomic DNA was extracted using the NucleoBond HMW DNA Kit (Macherey-Nagel, Düren, Germany) and purified using the DNeasy PowerClean Pro Cleanup Kit (Qiagen, Venlo, the Netherlands). Short DNA fragments were removed using the Short Read Eliminator XS Kit (PacBio, CA, USA). DNA fragments were then sheared to approximately 10–20 kb using a g-TUBE (Covaris, MA, USA). SMRTbell libraries were prepared using the SMRTbell Express Template Prep Kit version 2.0 (PacBio), and polymerase complexes were formed using the Binding Kit 2.2 (PacBio). Sequencing was performed on a PacBio Sequel IIe system. Subreads were generated by removing adapter sequences with SMRT Link version 11.0.0.146107 using default parameters. High-fidelity (HiFi) reads with quality scores of >20 were obtained for SY163 (20,110 reads) and SY183 (20,304 reads). Reads shorter than 1,000 bp were filtered out using Filtlong version 0.2.1 (4), resulting in 17,853 and 17,850 HiFi reads for SY163 and SY183, respectively. *De novo* assembly was performed with Flye version 2.9.1-b1780 using default settings (5), resulting in single circular chromosomes for both strains. Genome coverages were 51.0× for SY163 and 49.8× for SY183, with *N*_50_ values corresponding to the full chromosome lengths. The completeness of the genome assemblies was evaluated using CheckM version 1.2.2 with Enterobacterales-specific marker sets, achieving 99.9% completeness rates for both strains (6). Annotation was performed using DFAST version 1.3.3 with the rotate option to place the dnaA gene first (7), employing MetaGeneAnnotator v2008/08/19 for coding sequences (8), Barrnap version 0.8 for rRNA genes (9), and ARAGORN version 1.2.38 for tRNA genes (10). Default parameters were used for all software.

The genome of strain SY163 consists of 4,915,045 bp with a G + C content of 59.1%, encoding 4,431 protein-coding genes, 22 rRNA genes (5S, 16S, and 23S), and 87 tRNA genes. Strain SY183 has a genome size of 4,978,521 bp with a G + C content of 59.1%, containing 4,476 protein-coding genes, 22 rRNA genes, and 89 tRNA genes.

Average nucleotide identity analysis using Pyani version 0.2.12 (ANIm) (11) revealed that SY163 and SY183 were 98.6% and 99.1% identical, respectively, to the type strain of *Serratia rubidaea*, FDAARGOS_926 (GCA_016026735.1). These findings support future studies to identify antifungal genes and develop biocontrol methods using antifungal bacteria symbiotic with plants.

## Data Availability

The sequencing data of *Serratia rubidaea* strains SY163 and SY183 are available in the DDBJ Sequence Read Archive under accession numbers DRX604860 and DRX604861. Their complete genome sequences have been deposited under BioSample accession numbers SAMD00853225 (SY163) and SAMD00853224 (SY183), and genome assembly accession numbers AP038874 and AP038873.
